# Prevalence of geriatric syndromes in frail patients and mortality risks

**DOI:** 10.3389/fmed.2023.1165709

**Published:** 2023-07-06

**Authors:** O. N. Tkacheva, N. V. Sharashkina, K. A. Eruslanova, S. N. Lysenkov, A. A. Reshetova, L. V. Matchekhina, A. K. Ilyushchenko, N. K. Runikhina

**Affiliations:** ^1^Russian Gerontology Clinical Research Center, Ministry of Healthcare of the Russian Federation, Pirogov Russian National Research Medical University, Moscow, Russia; ^2^Faculty of Biology, Lomonosov Moscow State University, Moscow, Russia; ^3^State Budgetary Healthcare Institution "City Outpatient Clinic No. 109 of the Moscow Health Department", Moscow, Russia

**Keywords:** malnutrition, anemia, hearing impairment, geriatric syndromes, frailty

## Abstract

The prevalence of older people in Russian population increases rapidly. Therefore, the concept of healthy aging is becoming crucial in Russia and all over the world, and thus disability prevention is one aspect of this issue.

**Aim:**

To assess a possible association between geriatric syndromes, comorbidities, and mortality rate among frail patients who receive home medical care in Moscow.

**Materials and methods:**

The study included 450 patients with home medical care provided by the State Budgetary Healthcare Institution “Diagnostic Center No. 3 of the Moscow Health Department” from June 2019 to April 2021. Physical health, functional, cognitive, social and emotional statuses were evaluated by comprehensive geriatric assessment (CGA). The mortality rate after 1 year was assessed.

**Results:**

The all-cause case mortality rate in patients during the observation period was 22.4%. There was no difference in age and comorbidities in survivors and deceased patients, but the latter group had more geriatric syndromes. The association between risks of mortality and anemia and some geriatric syndromes, such as malnutrition and hearing impairment, total dependence (Barthel index less than 60) was observed.

## Introduction

Population aging requires society to adjust by ensuring new services and assistance for older adults. As for now about eight million people over working age in Russia are disabled, 6.4% of all people who receive social support need home care, and 62.2% are completely bedridden (1, 2). According to estimates of Federal State Statistics Service (Rosstat), the number of people over working age will increase further by 2031 in Russia, reaching the number of 42.7 million people (28.7%). The prevalence of people over 70 is growing most rapidly: the number of people in this age rose twofold over the past 20 years and accounts for 6% of the general population of Russia (3–5). It is evident that with population aging, the number of people who lack autonomy and need help and care will rise, and well as the burden on social, medical institutions.

Older patients have decreased physical and functional activity, adaptive and rehabilitation abilities, and higher risk of poor outcomes: risk of hospitalization is elevated in 1.2–1.8 times, the risk of physical impairment—in 1.6–2.0 times, mortality risk—in 1.8–2.3 times, risk of falls and fractures—in 1.2–2.8 times (2). In this regard, the analysis of morbidity and mortality in the most vulnerable groups of older adults is of particular interest. Among these groups are home medical care patients, including people with limited mobility and self-care, severe disabilities and geriatric syndromes: frailty, dementia, visual and hearing impairments.

Home medical care in Moscow outpatient clinics was established in 2017 to regularly provide primary health care to patients with limited mobility. Since 2019, geriatricians have joined a multidisciplinary team to provide home medical care. Doctors who work in the service also started retraining in geriatric medicine. Home care doctors began to use geriatric clinical practice guidelines.

We analyzed the data from the patient registry, including complex results of CGA to assess a possible association between geriatric syndromes, comorbidities, and case mortality rate among frail patients who receive home medical care in Moscow.

## Materials and methods

**Figure 1:**
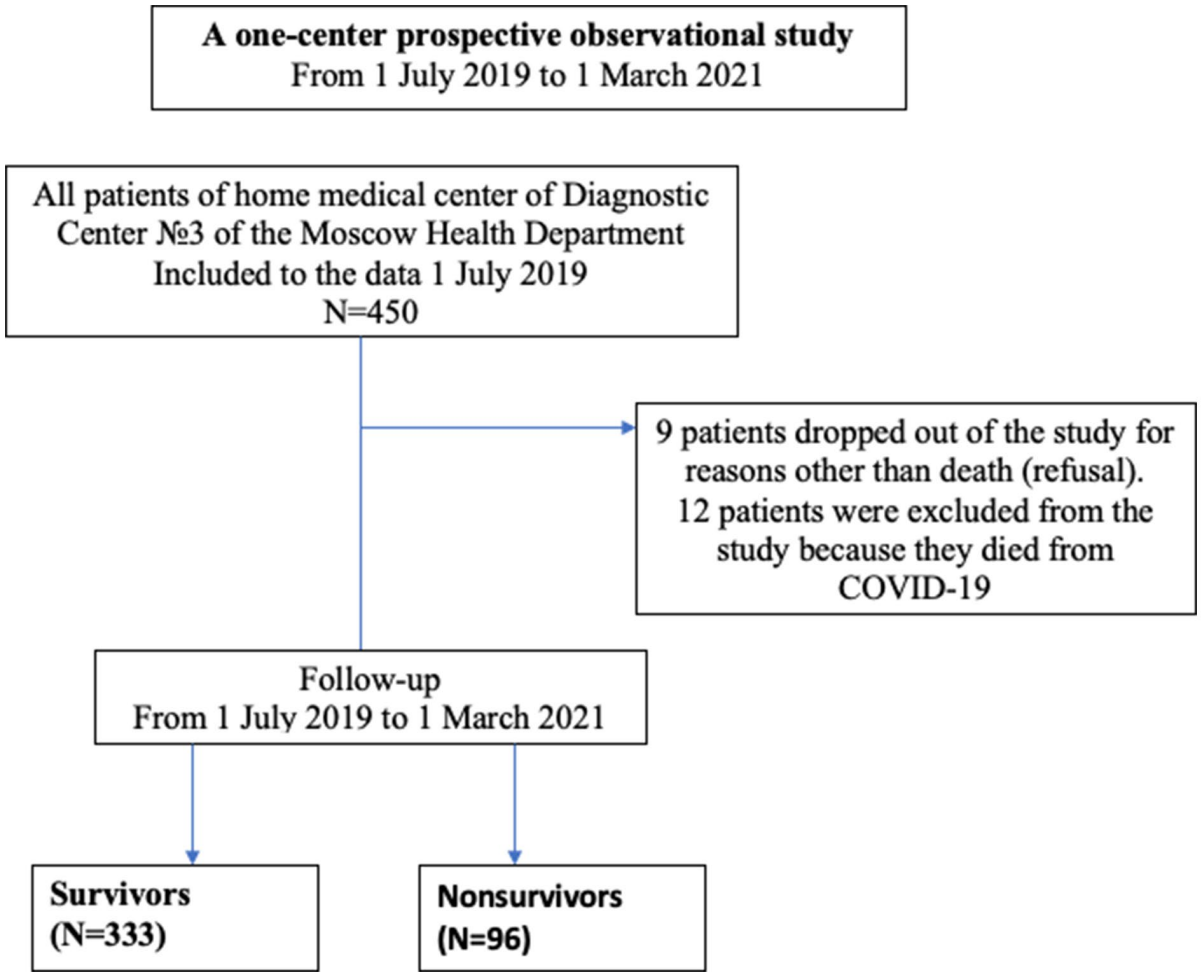
Patient flow chart template.

The observation cohort study included 450 frail patients who received home medical care by the State Budgetary Healthcare Institution “Diagnostic Center No. 3 of the Moscow Health Department” from July 2019 to March 2021 (Figure 1).

Exclusion criteria:

1. Patients <60 years of age.
2. Patients who did not received home medical care.

The information was taken from the patient registry, individual medical records, and comprehensive geriatric assessment (CGA) data collected before patient follow-up. Physical health, functional, cognitive, social status, and depression were assessed using CGA data (4, 6). Complex geriatric assessment included past medical history, geriatric scales and questionnaires [mini-mental state examination (MMSE), geriatric depression scale (GDS 15)], instrumental activity of daily living (IADL), the Barthel index (BI), nutritional assessment scale (MNA), insomnia rating scale (ISI) (7, 8). Case mortality rates were calculated from the beginning of the study to March 2021.

Nine patients dropped out of the study for reasons other than death (refusal). Another 12 people were excluded from the study because the cause of death was COVID-19, as this factor could affect survival differently and it was present only during a part of the study period. However, these 21 patients were included into survival analysis as censored observations.

A patient examination included:

1. Muscle strength assessment by hand dynamometry. Each patient was assessed for upper-limb strength in the standing position by means of the handgrip strength measurement of the dominant hand.
2. Each person was measured for height and weight and based on the obtained data, and then the Body Mass Index (BMI) kg/m^2^ was determined.
3. Measurement of blood pressure (BP), pulse rate.

All tests, scales and questionnaires used in the study are presented in the clinical guidelines “frailty” (4, 6).

The presence of the following geriatric syndromes was assessed: (1) frailty; (2) depression; (3) malnutrition; (4) urinary incontinence; (5) cognitive impairment; (6) functional dependence (7) falls (for the previous year); (8) vision impairment (9) hearing impairments (10) chronic pain (11) pressure ulcers.

The statistical analysis was performed using STATISTICA 10 software (StatSoft Inc., United States). Continuous variables are described as means and standard deviations, and categorical as frequencies and proportions (*n* and %). Mann–Whitney *U*-test was used for intergroup comparisons of quantitative variables, and two-tailed Fisher’s exact test was used for qualitative variables. Differences were considered statistically significant at *p* < 0.05.

Cox regression with stepwise selection of variables was used to identify factors that independently affect mortality in the study period. The model input was those variables that showed significant differences between the deceased patients and the survivors. Patients who dropped out of the study for reasons not related to death, as well as those who died from COVID-19 were treated as censored observations. The Cox regression result is reported as a hazard ratio with a 95% confidence interval.

As part of a comprehensive geriatric assessment, the presence of frailty syndrome was assessed using a short battery of physical functioning tests, the presence of frailty was indicated at short physical performance battery (SPPB) less than 7 points; baseline functional status was assessed using the Barthel scale, participants with a score of less than or equal to 95 had functional dependence, a score of less than 60 points on this scale was regarded as total dependence. Cognitive status was assessed using a mini-mental state examination (MMSE) mini-mental status assessment scale, a score of less than 24 points on this scale was regarded as cognitive impairment, dementia was determined by the conclusion of geriatric and neurologist. The mini nutritional assessment (MNA) was used to assess nutritional status, malnutrition was considered as an MNA score <17 points, the risk of malnutrition was less than 23.5 points. The presence of depression was assessed according to the results of the geriatric depression risk scale (GDS-15), a score of 5 or more was regarded as depression. The insomnia severity index (ISI) was used to assess insomnia, insomnia was considered as an ISI score more than 7. The risk of falls was assessed by a 12 months history of falls, if the participant fell 1 or more times in the last year, then the risk of falls was assessed as high. To assess the presence of polypharmacy, the number of drugs taken regularly (daily use of 5 or more drugs) was assessed. Assessment of the presence of chronic pain syndrome was carried out using the VAS visual analog scale, pain for lasts more than 3 months. Data on such geriatric syndromes as urinary incontinence, visual impairments, hearing impairments, pressure ulcers was taken from past medical history.

The hemoglobin level was used to assess anemia. Results below 12 g/dL in females and below 13 g/dL in males indicated anemia (8).

## Results

A one-center prospective observational study included 450 patients (29.7% men) aged 65 to 107 years. The mean age (±SD) was 81.0 ± 9.0 years, 70.3% were women. 354 (78.7%) of patients had assistance of social worker, 41 (9.1%) were wheelchair-bidden patients and 53 (11.8%) were bedridden patients. Immobilization, lying in bed for extended periods, contributed to the development and appearance of pressure ulcers in 10.0% of patients, and 3.1% of patients had urinary catheter and intestinal stoma.

We found high prevalence of arterial hypertension—433 (96.2%), coronary heart disease—391 (86.9%) and cerebrovascular disease—430 (95.6%); as well as 108 (24.0%) patients suffered from diabetes mellitus, 54 (17.8%) patients had cancer, and 74 (16.4%) patients had atrial fibrillation.

The most common geriatric syndromes among patients were urinary incontinence—262 (58.2%), functional dependence (Barthel scale less than 60 points)—231 (51.3%), dementia—(MMSE ≤24)—199 (44.2%), falls—190 (42.2%), visual impairment—401 (89.1%), and hearing impairment—143 (31.2%), fractures—112 (24.9%) and malnutrition—[MNA less than 17.5–8 (1.8%)].

The MMSE scores in the group ranged from 17 to 27 points. There were high scores on the geriatric depression scale, GDS-15 (mean of 11.8 ± 1.5), and insomnia scale, ISI (mean of 12.3 ± 1.4). After 1 year of observation, 64 people (33.2%) out of 193 patients with dementia (MMSE ≤24), who remained under observation, died, and in the group of patients with non-dementia cognitive impairment (MMSE >25) 54 out of 248 people (21.8%) died. These differences were statistically significant (*p* = 0.009). Patients with dementia and patients with non-dementia cognitive impairment were comparable in terms of demographic and anthropometric characteristics, had a comparable level of blood pressure, patients with dementia were distinguished by a greater severity of chronic pain syndrome, depression, sleep disorders, malnutrition, anemia (*p* < 0.05).

During the observation period, 96 out of 429 patients died. No significant differences in age and sex were found between deceased and survivors (Table 1).

**Table 1:** Comparison of survivors and deceased patients.

	Survivors (*n* = 333)	Deceased patients (*n* = 96)	*p*-value
Age	81,0 ± 9,0	81,4 ± 8,8	0,68
Sex, women, *n* (%)	260 (78%)	68 (70%)	0,17
Cerebrovascular diseases, *n* (%)	319 (96%)	93 (97%)	0,77
Coronary heart disease, *n* (%)	293 (88%)	78 (81%)	0,09
Arterial hypertension, *n* (%)	320 (96%)	93 (97%)	1
Atrial fibrillation, *n* (%)	61 (18%)	10 (10%)	0,08
Diabetes mellitus, *n* (%)	87 (26%)	19 (20%)	0,23
Gastrointestinal diseases, *n* (%)	65 (20%)	14 (15%)	0,30
Respiratory diseases, *n* (%)	22 (7%)	8 (8%)	0,65
Cancer, *n* (%)	39 (12%)	12 (13%)	0,86
Systolic blood pressure, mm Hg	133,0 ± 13,0	140,9 ± 12,5	<0,01
Diastolic blood pressure, mm Hg	85,8 ± 9,4	89,8 ± 7,4	<0,01
The Barthel scale	55,0 ± 13,6	48,2 ± 16,7	<0,01
MMSE	23,6 ± 2,2	22,6 ± 2,5	0,64
GDS 15	11,6 ± 1,4	12,4 ± 1,4	<0,01
ISI	12,0 ± 1,3	13,0 ± 1,5	<0,01
Visual analogue scale (VAS)	1,1 ± 1,1	1,6 ± 1,3	<0,01
MNA	22,7 ± 1,2	21,8 ± 1,9	<0,01
Dementia	195 (59%)	70 (73%)	0,01
Hand dynamometry, kg	9,8 ± 2,3	9,6 ± 2,2	0,56
BMI, kg/m^2^	24,2 ± 2,3	23,4 ± 2,1	<0,01
Anemia	94 (28%)	45 (47%)	<0,01
Malnutrition (МNA <17.5) and risk of malnutrition (МNA 17.5–23)	0	7 (7 %)	<0,01
Visual impairments, *n* (%)	293 (88%)	89 (93%)	0,26
Hearing impairments, *n* (%)	94 (28%)	42 (44%)	0,01
Falls, *n* (%)	138 (41%)	44 (46%)	0,48
Fractures, *n* (%)	73 (22%)	34 (35%)	0,01
Urinary incontinence, *n* (%)	195 (59%)	55 (57%)	0,91

**Figure 2:**
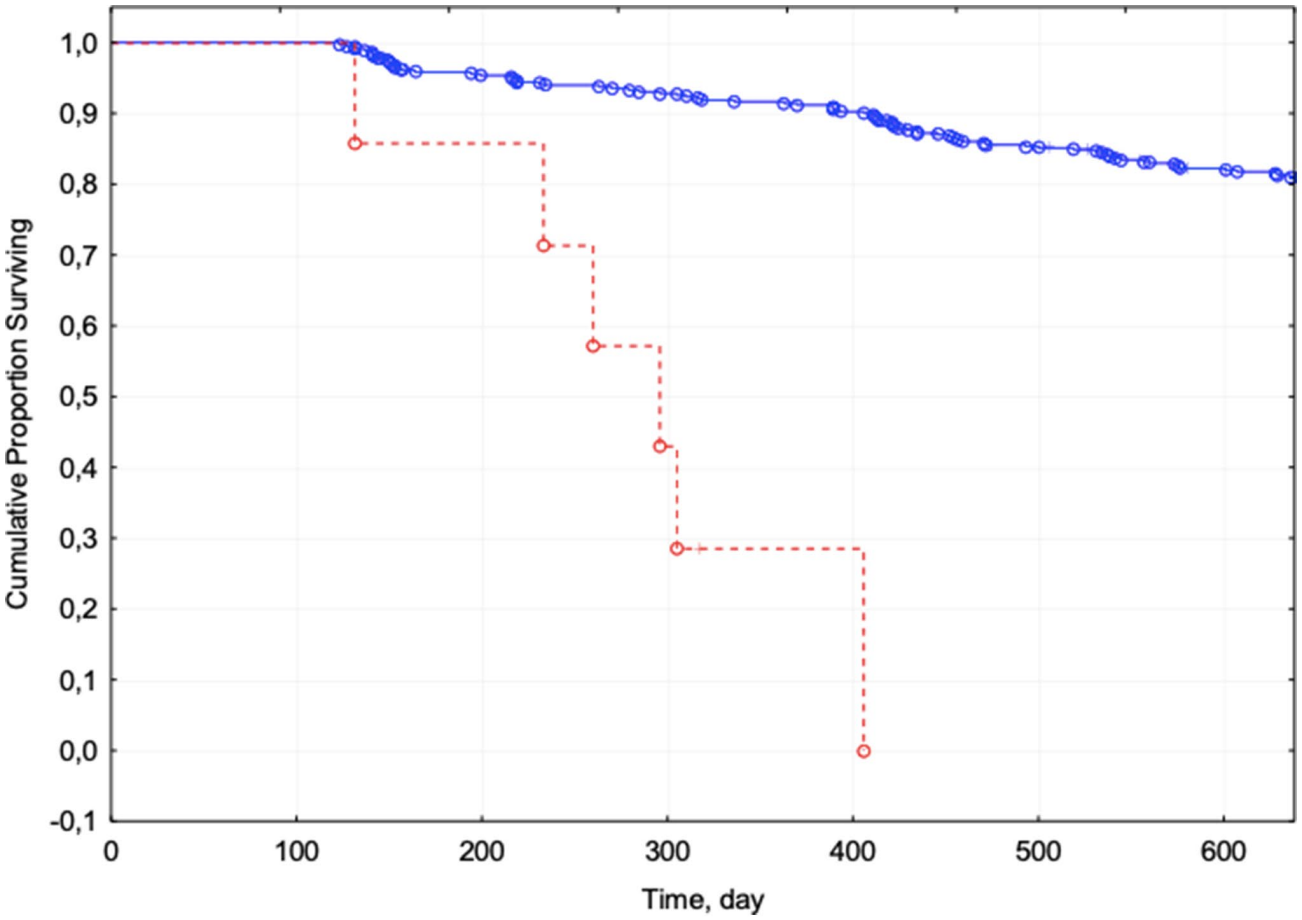
Kaplan–Meier curves for patients with malnutrition (red) and without malnutrition (blue). Complete observations are marked with circles, censored ones with crosses.

The presence of dementia, anemia, malnutrition, and chronic pain significantly differed in the group of survivors and deceased patients; patients with sleep disorders, depression, and a history of fractures had higher mortality risk. Cox regression with stepwise selection of variables, however, showed a significant effect of only one factor, malnutrition, increasing the mortality risk by 1,206 times (95% CI 463–3,142; Figure 2).

Since malnutrition was noted in only a few patients we also performed a Cox regression without considering this feature. In frail patients, anemia and hearing impairment increase the mortality risk by more than 1.5 times, and the presence of total dependence (BI <60)—by almost 2.5 times (Table 2).

**Table 2:** Factors independently associated with poor outcomes.

Parameters	Hazard ratio (HR)	95% confidence interval for HR	*p*-value
Anemia	1,62	1,03–2,54	0,04
Total dependence (BI <60)	2,35	1,12–4,96	0,02
Hearing impairments	1,63	1,06–2,52	0,03

Thus, a complex of factors independently associated with mortality in home medical care patients was identified: malnutrition, anemia, total dependence (BI <60) and hearing impairment.

## Discussion

A multivariate analysis of all parameters (chronic diseases and geriatric syndromes) demonstrated that independent risk factors for mortality in home medical care patients are malnutrition, total dependence (BI <60), anemia and hearing impairment. Primary risk factors for poor outcomes and death in patients in our study were not associated with age or comorbidities.

Earlier studies of diseases associated with poor outcomes demonstrated that high mortality risk in the older patients is associated with chronic heart failure, cancer, chronic kidney disease, chronic obstructive pulmonary disease, stroke and obesity (9, 10). Either muscle strength is an important factor influencing health in frail patients, muscle strength is related to mental and physical quality of life (11). Recent studies have also suggested that severe dementia and functional dependence, in addition to age and multimorbidity, were associated with increased mortality risk (10, 12, 13). The prevalence of polypharmacy is high in home medical care patients, use of multiple medications increased with the number of co-existing diseases and the presence of specific symptoms (14). While some groups of drugs, for example ACE-inhibition, can have a positive effect and improve the quality of life in older adults (15, 16).

Malnutrition was one of the most significant factors associated with mortality in our study. According to meta-analyses included 240 studies the prevalence of malnutrition (using MNA) is 6.0% in outpatients, 8.7% in home-care services, 22.0% in hospital, 17.5% in nursing homes, 17.5 28.7% in long-term care (17). In our study, the prevalence of malnutrition syndrome was 7%. All patients with malnutrition (less than 17.5 points according to MNA) were in the group of deceased patients. It should be noted that, according to multivariate analysis, malnutrition turned out to be one of the independent predictors; the correlation of the degree of malnutrition with survival gives grounds for using nutritional status as one of the leading predictive criteria for poor outcomes in home medical care patients. In older patients, the detection and timely correction of malnutrition play a significant role since there is a close relationship between malnutrition and adverse outcomes—an increase in the frequency of infections and pressure ulcers, the length of stay in the hospital, and the frequency of repeated hospitalizations (18, 19). Malnutrition is associated with an increased risk of all-cause mortality. Assessment of nutritional status and correction of malnutrition is an essential step in the treatment of this cohort of patients.

Anemia also is now recognized as a risk factor for several adverse outcomes in older adults, including hospitalization, increased morbidity and mortality risk (20, 21). More than 10% of adults ≥65 years of age surfer from anemia (hemoglobin <12 g/dL in women and <13 g/dL in men) (22). With the acceptance of importance of anemia treatment in the general population, guidelines have been published for detecting, evaluating, and managing anemia in older patients. According to multivariate analysis, in our study, anemia increased the mortality risk by 1.62 times.

Another finding of our study revealed that the higher mortality risk was associated with hearing impairment. According to Miyawaki, hearing impairment was associated with increased mortality in middle-aged and older adults in Japan (23). Authors supposed that it could be related to the social isolation of patients with hearing impairments and a corresponding decrease in cognitive function, physical activity, and mental health.

It is estimated that the total number of people who need long-term care will stand at 10.3 million by 2025 in Russia, including 4.65 million who will need home medical care (a threefold increase from 2017) (1, 3, 5).

The main factors limiting the quality of life of older people in Russia are associated with poor health, low life expectancy in older age, Obstacles for volunteers and social workers, problems with continuous education and physical activity (5). Although the life expectancy in Russia has increased, the country consistently ranks last on the corresponding among European countries. It has a significant gender difference in life expectancy, life expectancy at the age of 60 in Russia is at 15.5 years for men and 21 years for women. Measures to prevent and treat diseases, improve older people health, and increase life expectancy should be based on the health care system and the availability of drugs, safe living and working conditions, environmental well-being, and the efforts of older people to maintain health. In the last 5–7 years of life, the older adults lose autonomy due to chronic diseases and frailty. As a result, they need help and long-term care (1, 5, 8).

### Study strengths and limitations

The study has several advantages. To our knowledge, this is the first study that assessed possible associations between geriatric syndromes, comorbidities, and mortality rate among patients who receive home medical care in Moscow. The study population was well defined, and there was a complete database on overall mortality. Our findings should be considered within certain limitations. First, we did not take into account the causes of death. Second, the participants of the study were home medical care patients, who are usually more frail than people who live in the community.

## Conclusion

Analyzing the available research data makes it possible to mark the path for further investigations. Bearing in mind the number of comorbidities we have to face in geriatric patients, the multidisciplinary approach should be implemented in the daily clinical routine. A comprehensive geriatric assessment in the home medical care service allows to identify patients with risk factors and predisposed to adverse outcomes. The data obtained in our study might be used to upgrade the examination procedure, treatment, and long-term care in Moscow home medical care service.

## Data availability statement

The raw data supporting the conclusions of this article will be made available by the authors, without undue reservation.

## Ethics statement

The studies involving human participants were reviewed and approved by the Russian Gerontology Research and Clinical Centre Ethics Committee. The patients/participants provided their written informed consent to participate in this study.

## Author contributions

All authors listed have made a substantial, direct, and intellectual contribution to the work and approved it for publication.

## Conflict of interest

The authors declare that the research was conducted in the absence of any commercial or financial relationships that could be construed as a potential conflict of interest.

## Publisher’s note

All claims expressed in this article are solely those of the authors and do not necessarily represent those of their affiliated organizations, or those of the publisher, the editors and the reviewers. Any product that may be evaluated in this article, or claim that may be made by its manufacturer, is not guaranteed or endorsed by the publisher.

## Supplementary material

The Supplementary material for this article can be found online at: https://www.frontiersin.org/articles/10.3389/fmed.2023.1165709/full#supplementary-material
