# Supplementary material for: Prevalence of geriatric syndromes in frail patients and mortality risks
Source: Front Med (Lausanne). 2023 Jul 6;10:1165709. doi: 10.3389/fmed.2023.1165709 (PMC10357963; doi:10.3389/fmed.2023.1165709)
Supplement: Supplementary file 1 [file Data_Sheet_1.docx]

Supplementary Material

Article Title

Prevalence of geriatric syndromes in frail patients and mortality risks

**TKACHEVA O.N.1, SHARASHKINA N.V.1*, ERUSLANOVA KA1, LYSENKOV S.N.1,3**

**RESHETOVA A.A.2 , MATCHEKHINA L.V.1, ILYUSHCHENKO A.K.1 RUNIKHINA N.K.1**

*** Correspondence:** SHARASHKINA N.V.

sharashkina@inbox.ru

# Supplementary Figures and Tables

Supplementary Figures
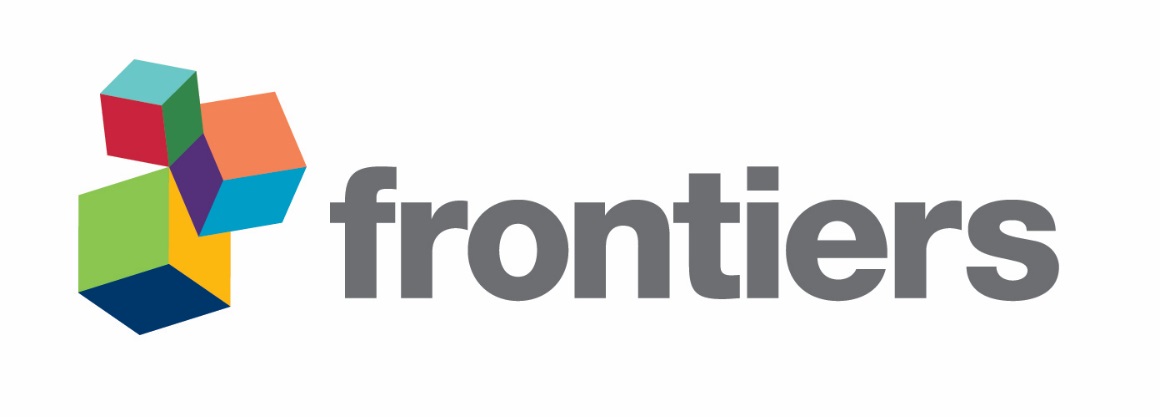
Supplementary Figure 1.


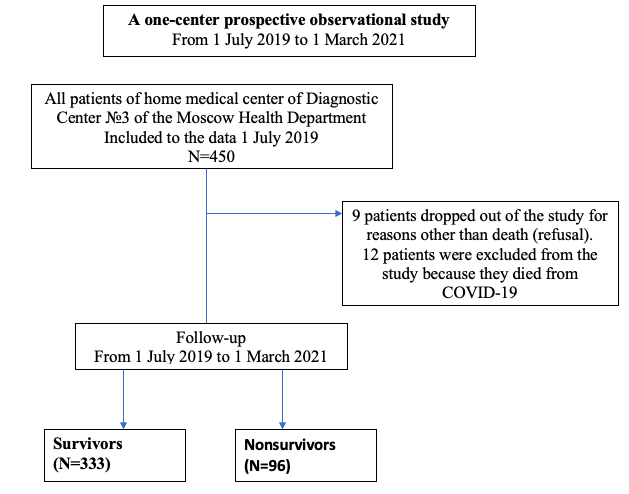


**Fig.1 Patient Flow Chart Template**

Supplementary Figure 2.

##
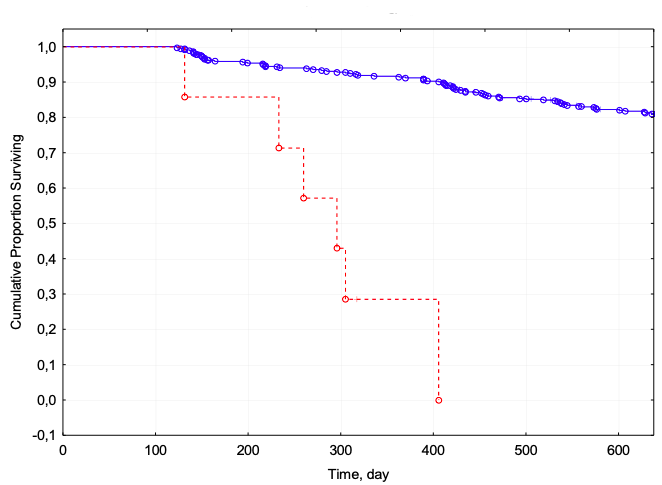


## Fig.2. Kaplan-Meier curves for patients with malnutrition (red) and without malnutrition (blue). Complete observations are marked with circles, censored ones with crosses.

**Table 1**

**Table 1**

**Comparison of Survivors and Deceased patients**

|  | **Survivors**  **(n=333)** | **Deceased patients**  **(n=96)** | **P** |
| --- | --- | --- | --- |
| Age | 81,0±9,0 | 81,4±8,8 | 0,68 |
| Sex, women, n (%) | 260 (78%) | 68 (70%) | 0,17 |
| Cerebrovascular diseases, n (%) | 319 (96%) | 93 (97%) | 0,77 |
| Coronary heart disease, n (%) | 293 (88%) | 78 (81%) | 0,09 |
| Arterial hypertension, n (%) | 320 (96%) | 93 (97%) | 1 |
| Atrial fibrillation, n (%) | 61 (18%) | 10 (10%) | 0,08 |
| Diabetes mellitus, n (%) | 87 (26%) | 19 (20%) | 0,23 |
| Gastrointestinal diseases, n (%) | 65 (20%) | 14 (15%) | 0,30 |
| Respiratory diseases, n (%) | 22 (7%) | 8 (8%) | 0,65 |
| Cancer, n (%) | 39 (12%) | 12 (13%) | 0,86 |
| Systolic blood pressure, mm Hg | 133,0±13,0 | 140,9±12,5 | <0,01 |
| Diastolic blood pressure, mm Hg | 85,8±9,4 | 89,8±7,4 | <0,01 |
| The Barthel Scale | 55,0±13,6 | 48,2±16,7 | <0,01 |
| MMSE | 23,6±2,2 | 22,6±2,5 | 0,64 |
| GDS 15 | 11,6±1,4 | 12,4±1,4 | <0,01 |
| ISI | 12,0±1,3 | 13,0±1,5 | <0,01 |
| Visual Analogue Scale (VAS) | 1,1±1,1 | 1,6±1,3 | <0,01 |
| MNA | 22,7±1,2 | 21,8±1,9 | <0,01 |
| Dementia | 195 (59%) | 70 (73%) | 0,01 |
| Hand dynamometry, kg | 9,8±2,3 | 9,6±2,2 | 0,56 |
| BMI, kg/m2 | 24,2±2,3 | 23,4±2,1 | <0,01 |
| Anemia | 94 (28%) | 45 (47%) | <0,01 |
| Malnutrition (МNA <17.5) | 0 | 7 (7 %) | <0,01 |
| Visual impairments, n (%) | 293 (88%) | 89 (93%) | 0,26 |
| Hearing impairments, n (%) | 94 (28%) | 42 (44%) | 0,01 |
| Falls, n (%) | 138 (41%) | 44 (46%) | 0,48 |
| Fractures, n (%) | 73 (22%) | 34 (35%) | 0,01 |
| Urinary incontinence, n (%) | 195 (59%) | 55 (57%) | 0,91 |

**Table 2**

**Table 2. Factors independently associated with poor outcomes**

| Parameters | Hazard ratio (HR) | 95% Confidence interval for HR | Р value |
| --- | --- | --- | --- |
| Anemia | 1,62 | 1,03-2,54 | 0,04 |
| Functional dependence (BI <60) | 2,35 | 1,12-4,96 | 0,02 |
| Hearing impairments | 1,63 | 1,06-2,52 | 0,03 |
